# Organisation of Multi-Mycotoxin Proficiency Tests: Evaluation of the Performances of the Laboratories Using the Triple A Rating Approach

**DOI:** 10.3390/toxins13090591

**Published:** 2021-08-24

**Authors:** Emmanuel K. Tangni, Bart Huybrechts, Julien Masquelier, Els Van Hoeck

**Affiliations:** Organic Contaminants and Additives, Scientific Directorate of Chemical and Physical Health Risks, Sciensano, Leuvensesteenweg 17, 3080 Tervuren, Belgium; bart.huybrechts@sciensano.be (B.H.); Julien.Masquelier@sciensano.be (J.M.); Els.VanHoeck@sciensano.be (E.V.H.)

**Keywords:** proficiency testing, performances, triple A, reference materials, mycotoxins, cereals

## Abstract

In accordance with the International Standard Organization ISO 17043, two proficiency tests (PTs) for the simultaneous determination of aflatoxins (AFB_1_, AFB_2_, AFG_1_, AFG_2_); deoxynivalenol; fumonisins FB_1_, FB_2,_ and B_3_; ochratoxin A, the T-2 toxin; and the HT-2 toxin were conducted in 2019 and 2020 using cornflakes and rusk flours that were prepared in house. The homogeneity and the stability of these materials were verified according to the criteria laid down in ISO 13528 using randomly selected samples. Most of the targeted toxins were found to be homogenously distributed in both materials with no significant changes during the timescale of the PTs. Next, the materials were distributed to approximately 25 participating laboratories from Europe, Canada, and the United States. The obtained datasets were computed using robust statistics. The outliers were checked and removed, and the toxin concentrations were assigned as the consensus value of the results of the participants at Horwitz ratios <1.2. The z scores were generated for all mycotoxins, and the results were pooled to calculate the relative sum of squared z scores (SZ2) indexes and were clustered according to the triple A rating. Overall, at least 80% of the participating laboratories achieved good and acceptable performances. The most frequent categories assigned to good performances (SZ2 ≤ 2) were AAA (51%) and BAA (13%). Clusters of BBA + CBA (6%) included laboratories reporting acceptable z scores <90% of the total z scores for less than 90% or 50% of the mycotoxins targeted in the 2 matrices. The triple A rating seems to be appropriate in evaluating the performances of laboratories involved in multi-mycotoxin analyses. Accredited and non-accredited analytical methods achieved good and acceptable performances.

## 1. Introduction

The occurrence of mycotoxins is widespread throughout the world. The Food and Agriculture Organization (FAO) previously estimated that 25% of the global food crop could be contaminated with mycotoxins. More recent evaluation of the exceedances of the limits set by the European Union (EU) and *Codex Alimentarius* concluded that the occurrences of mycotoxins were much higher and reached up to 60–80% [1]. Furthermore, several studies have evidenced a higher frequency of finding more than one mycotoxin in food and feed due to the co-existence of fungi that simultaneously produce several mycotoxins [2,3]. The co-occurrence of mycotoxins and related interactive toxic effects, even at low concentrations, have raised concerns about the health hazards related to contaminated food and feed [4,5,6]. In addition, mycotoxin contaminated feed may release their toxic substances or their bio-transformed metabolites into animal tissue, offal, milk, and eggs and thus may obviously increase the consumer’s exposure [7]. Contamination removal via food processing has not been fully achieved [8]. Even in developed countries, agricultural products are still contaminated with mycotoxins, and climate changes have amplified and facilitated the appearance and dissemination of the regulated mycotoxins and the so-called “emerging mycotoxins” [9]. In Europe, regulatory maximum limits (MLs) or guideline levels were fixed for the presence of aflatoxin B_1_ (AFB_1_), total aflatoxins (AFB_1_+AFB_2_+AFG_1_+AFG_2_), citrinin (CIT), deoxynivalenol (DON), fumonisins B_1_ and B_2_ (FB_1_, FB_2_), patulin (PAT), ochratoxin A, T-2 toxin (T-2), the HT-2 toxin (HT-2), and zearalenone (ZEN) in food, food supplements, and feed commodities [10,11,12]. The development of analytical methods is a prerequisite for the measurement of mycotoxin co-contamination in food and feed, for control purposes, and for maintaining regulatory safety compliance [13,14,15]. It also allows the performance of toxico-kinetic profiling studies [16]. Moreover, the marketability of food products is critically based on reliable measurements of food quality and safety requirements [17]. Reliable analytical results are therefore essential for legislation implementation, quality and safety assurance of food and feed, risk assessment, and worldwide trade transactions. In accordance with the recommendations of the International Standard Organization ISO 17025 [18] and the EC regulations [19], all laboratories involved in official control analyses must provide evidence of their analytical competence through their successful participation in proficiency testing (PT). International PT providers such as the Food Analysis Performance Assessment Scheme (FAPAS), Bureau Interprofessionnel d’Etudes Analytiques (BIPEA), the Joint Research Centre (JRC-European Commission), Monitoring and Quality Assurance (MoniQA) have often proposed suitable PTs for single or a limited number of relevant mycotoxins. Few multi-mycotoxin PT schemes that include most of the regulated mycotoxins (AFB_1_, AFB_2_, AFG_1_, AFG_2_, OTA, DON, T-2, HT-2, ZEN, FB_1_ ,and FB_2_ exist [20]. Within the frame of the EC-funded MoniQA project, an international PT was based on spiked maize flours [20]. Moreover, participation in a PT enables the laboratories to detect and remedy shortcomings in their procedures [21,22]. If suitable multi-analyte reference materials are consequently needed, as stated by Solfrizzo et al. [20] and Tangni et al. [23], then adequate methodology for acknowledging the overall laboratory performances with several z scores generated for the targeted compounds and merits of the matrices to be applied [24]. The sum of ranking differences (SRD), principal component analysis (PCA), hierarchical cluster analysis, Youden plots, rescaled sum of z scores (RSZ), relative laboratory performance (RLP), sum of the squared z scores (SZ2), and the triple A approaches have already been applied for comparing the multi-analyte results reported during proficiency tests for polycyclic aromatic hydrocarbons [25] and pesticide residues [24,26]. Guidelines, tools, and results for the performance evaluation of analytical methods intended for the quantitative and semi-quantitative determination of multi-mycotoxins are often based on the individual z scores obtained by participating laboratories [23,27,28,29]. Recently, FAPAS organized a multi-mycotoxin in oat flour and stated that the consideration of a set or sequence of z scores over time provides more useful information than a single z score [29]. The present study aimed at organizing two international proficiency tests and at assessing the performances of the participating laboratories by means of combining the well-established z score values using the triple A approach against the relative sum of squared z scores index.

## 2. Results and Discussion

### 2.1. PT Test Materials

Cornflakes (maize product) and rusk (wheat product) were incurred contaminated with several mycotoxins such as AFB_1_, AFB_2_, AFG_1_, AFG_2_, OTA, FB_1_, FB_2_, FB_3_, DON, T2, HT2, and ZEN at various concentrations. The homogeneity was evaluated (Table 1 and Table 2). It should be noted that the concentration of AFB_2_ and AFG_2_ in the cornflake material were very close to the LOQs of the laboratories, i.e., 0.1 µg/kg for AFB_2_ and 0.5 µg/kg for AFG_2_. Although ZEN is a relevant toxin for corn-based matrices, it was not homogeneously distributed in the test materials. In the rusk test material, AFG_2_, FB_2,_ and FB_3_ were also present at very low concentrations: <0.5 µg/kg for AFG_2_ and 100 µg/kg for FB_2_ and FB_3_.

In accordance with the requirements of IUPAC [21] and ISO 13528 [30], the two candidate materials were adequately homogenous enough to run and were allowed the assignment of the targeted mycotoxins concentrations. The condition for “sufficient homogeneity” is that the true between-sample variability (σ_sam_) does not exceed 0.3 × σ_p_. The standard deviation (SD) of the 12 averaged results includes contributions from the between-sample variability and from the analytical variability s_an_; therefore, if this SD is <0.3 × σ_p_, then the material is certainly sufficiently homogenous; otherwise (>0.3 × σ_p_), the critical value test was applied.

Afterwards, the stability of the materials was also evaluated. The results are given in Table 3, illustrating that both PT materials were found to be adequately stable at +4 °C, as recommended.

### 2.2. Participants

**Figure 1:**
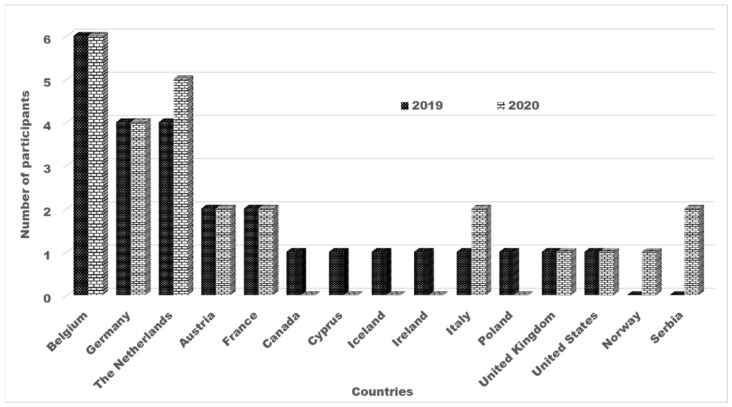
Countries of the participating laboratories in 2019 (*n* = 25) and in 2020 round (*n* = 26).

In 2019, 25 laboratories subscribed to participate in the PT (Figure 1), but one participant did not submit results within the requested deadline, and one laboratory reported results using two different methods. In total, 25 results were used for the robust statistics. Of the participating laboratories, nineteen laboratories were ISO 17025 accredited. The participants were from Europe, the United States, and Canada (Figure 1). In 2020, 26 laboratories (20 of which used ISO 17025 accredited methods) participated in the PT and reported their results. They were from Europe and the United States, as summarized in Figure 1.

### 2.3. Analytical Methods Used by the Participating Laboratories

In these PTs, all of the laboratories used liquid chromatography in combination with mass spectrometry LC-MS, except for two participants, who used fluorescence detection after immunoaffinity cleanup for the analysis of AFLs and OTA. ISO 17025-accreditation was obtained for the analytical methods used by 19 laboratories in 2019-PT against 20 laboratories in 2020-PT.

### 2.4. Assigned Values and Laboratory Performance Expressed as z Scores and ζ Scores

**Figure 2:**
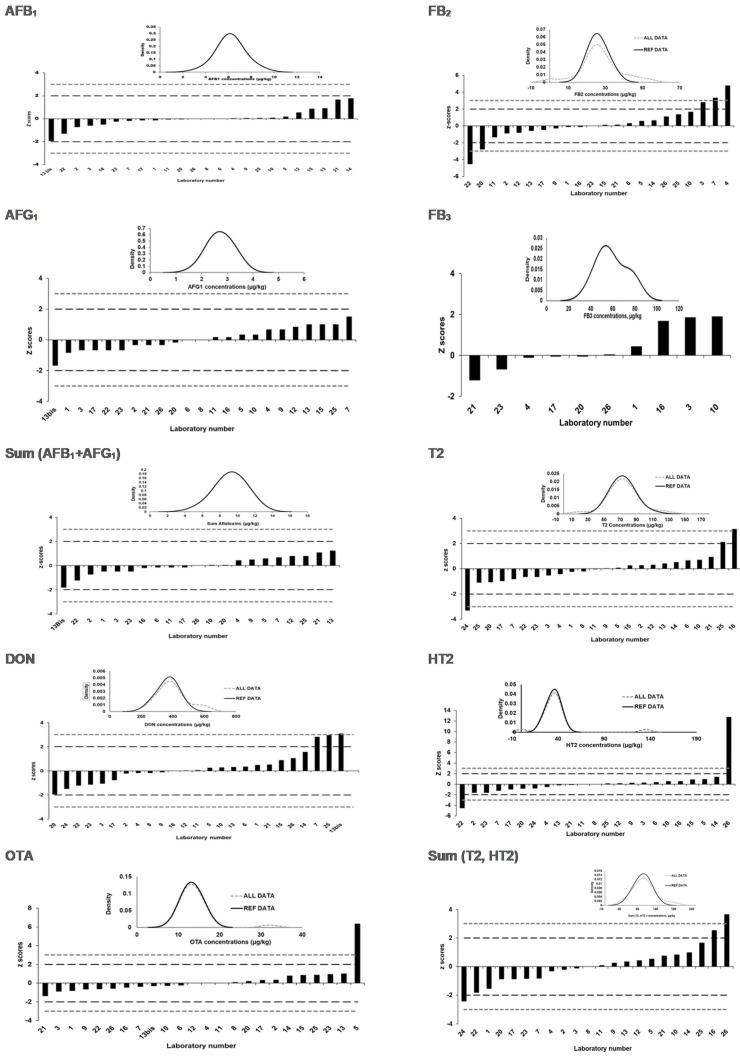
Kernel density plots in assigning reference values and graphs of the laboratories’ z scores for AFB_1,_ AFG_1,_ sum (AFB_1_, AFG_1_)_,_ DON, OTA, FB_2_, FB_3_, T2, HT2, and sum (T2, HT2) in 2019-PT materials.

**Figure 3:**
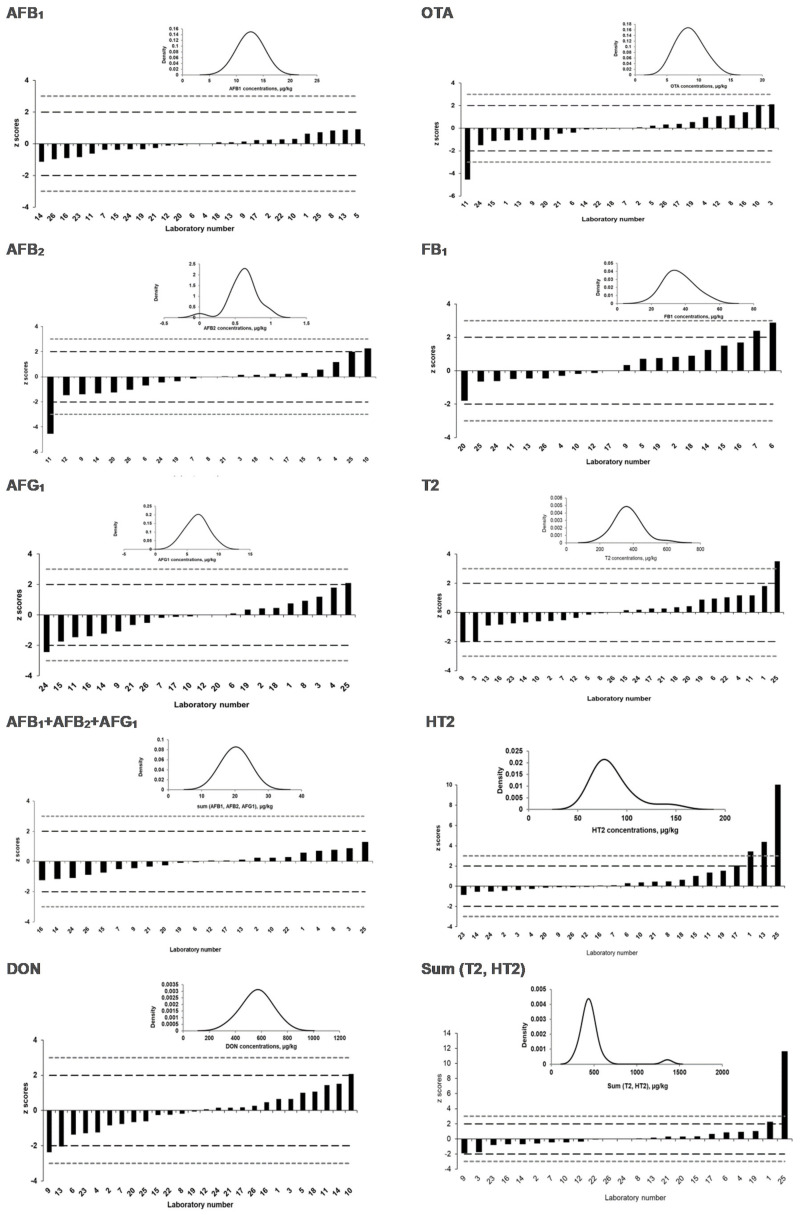
Kernel density plots in assigning reference values and graphs of the laboratories’ z scores for AFB_1,_ AFB_2,_ AFG_1_, sum (AFB_1,_ AFB_2,_ AFG_1_)_,_ DON, OTA, FB_1_, T2, HT2, and sum (T2, HT2) in 2020-PT materials.

The Horwitz value is widely recognized as a fit-for-purpose criterion in proficiency testing and allowed the estimation of the satisfying central tendency with the satisfactory HorRat < 1.2, which is used for deriving a consensus value for the mass fractions of the targeted mycotoxins [21]. Table 4 and Table 5 present the consensus values, standard uncertainties, and relevant statistical parameters for both PT rounds. The consensus values are the result of the straightforward calculation of the median in which all participants had the same status, and outliers are excluded. Nevertheless, the disadvantages are that the consensus values are dependent of the participant’s results. A low number of participants may increase the uncertainty on the consensus with the consequence of lowering the corresponding z scores [21]. The resulting z scores and ζ scores for each mycotoxin are summarized in Table 4 and Table 5 for both PT rounds. Figure 2 and Figure 3 displayed individual z-score results and kernel density plots in assigning the reference values of the mycotoxins for the 2019-PT and 2020-PT materials.

In 2019, an assigned value was attributed to AFB_1_, AFG_1_, sum (AFB_1_, AFG_1_), DON, FB_2_, FB_3_, OTA, T2, HT2, and sum (T2, HT2). In contrast, no assigned value (Horrat > 1.2) was attributed to FB_1_ and sum (FB_1_, FB_2_).

AFB_2_ and AFG_2_ were present at very low levels, i.e., 0.1 µg/kg for AFB_2_ and 0.5 µg/kg for AFG_2_. For these toxins, no laboratory reported a false positive result (i.e., erroneous reported concentration above LOQs).

For all of the targeted compounds, most of the participants obtained acceptable z scores for all of the mycotoxins (i.e., 77.3% to 100% of the participants). However, unacceptable DON results were obtained by 12.0% of the participants. Questionable z scores were obtained by 4.2% to 9.1% of the participants for T2, FB2, and sum (T2, HT2). It should be noticed that under the “normal” and “Gaussian” distribution hypothesis, the percentages of questionable or unacceptable results should be approximately 5% and 0.25%, respectively [21]. The cause analyses pertaining to an unacceptable z score were not performed by the PT provider but was considered to be the responsibility of the participating laboratories.

**Figure 4:**
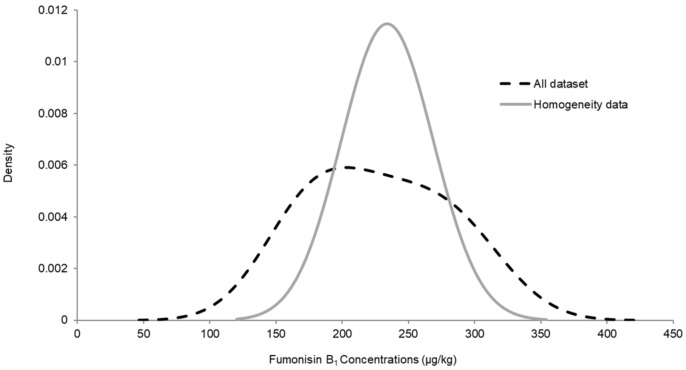
Kernel density plots for FB_1_ results (2019).

No assigned value could be attributed to FB_1_ due the very wide distribution of the results provided by participants (Figure 4). One possible hypothesis would be the existence of two populations within the results. A wide distribution of the Kernel-plot might be due to two peaks that are more or less merged. Most of the laboratories used an isotopically labelled standard for each individual toxin in order to compensate for matrix attenuation or matrix enhancement, the latter usually being the case with FBs. Therefore, an incorrect correction for this enhancement might quickly lead to an overestimation of the FBs concentrations. By comparing the data with the reported methodology, a small trend could be revealed that the laboratories that did not use a ^13^C-labelled internal standard reported higher values compared to the results obtained during the homogeneity study.

As FB_1_ was present at a much higher concentration than FB_2,_ the former dictates the results of the sum. As such, no assigned value could be attributed to the sum. Very few FB_3_ results (*n* = 10) with a large variation did not allow an assigned value for FB_3_ to be derived were reported by the participants. Note that there is no EU legislation for this component.

For all of the targeted compounds, acceptable ζ scores were achieved by 75.0% to 94.4% of the participants. However, 19.7% of the results were reported without measurement uncertainty (MU). Hence, no ζ score was calculated for these participants. Due to the importance of a correct estimation of the MU for compliance evaluation, more efforts should be devoted to the determination of the MU.

In the PT organized in 2020, assigned values were attributed to AFB_1_, AFG_1_, AFB_2_, the sum of the aflatoxins, DON, FB_1_, HT2, T2, sum (T2, HT2), and OTA, while no value was attributed to AFG_2_, FB_2_ and the sum of FB_1_ and FB_2_.

For both PTs, most of the laboratory performances were acceptable. Nevertheless, some extreme values for the ζ scores were observed and were often associated with relatively low claimed measurement uncertainties.

No value could be assigned for AFG_2,_ as it was present at a very low concentration (i.e., <0.5 µg/kg), and only nine participants reported a value. There was one laboratory, however, that reported a very high value of 8.3 µg/kg, which probably indicates a false positive result.

FB_2_ was also absent from the material, but no false positive results were reported here. Due to the absence of FB_2_, no assigned value could be attributed to the sum of FB_1_ and FB_2_.

**Figure 5:**
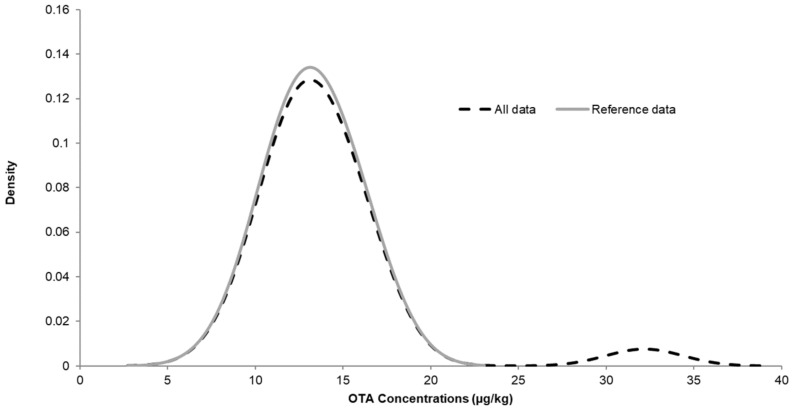
Kernel density plot for OTA.

The attribution of the assigned value for OTA was more complex, as the calculated Horrat value was 1.4, thereby exceeding the indicated maximum by IUPAC by 1.2. However, the Kernel density plot for the reference data clearly showed a symmetric and unimodal distribution (Figure 5), allowing an assigned value for this compound to be assigned.

### 2.5. Overall Laboratory Performance

#### 2.5.1. General Pattern of Laboratory Performances over Two-Year Period

Generated z scores for all regulated mycotoxins in the test materials were pooled to calculate the SZ2 indexes for both PT rounds. Table 6 presents the comparison of the percentages of the results obtained over a two-year evaluation according to the triple A rating and the SZ2 classification. The triple A rating approach takes into account the scope of the analytes, the acceptable z scores, and the false positive results [26].

Overall good and acceptable performances were achieved by 80% of the participants in 2019 and 89% of the participants in 2020. Therefore, participating laboratories have proven their competence in measuring the mycotoxins targeted in cornflake and rusk flours and may hence ascertain their accreditation to ISO 17025. Most frequent categories assigned to the participants in this cluster were AAA (57%) and BAA (13%). Note that laboratories in these two categories reported acceptable z scores >90% of the total number and no false positives. Most of the participating laboratories in the ABA category (10%) reported acceptable z scores that ranged between 50% and 90% of those of the total number of z scores. Underperformance was also noted for the laboratories in this cluster, with high unacceptable z scores or a high number of questionable z scores. The cluster of unacceptable performances includes categories BBA+CBA (6%). Some laboratories reported less than 50% of the mycotoxins targeted for the two matrices.

#### 2.5.2. Accreditation Status and Related Trends on Performance Evaluation

**Figure 6:**
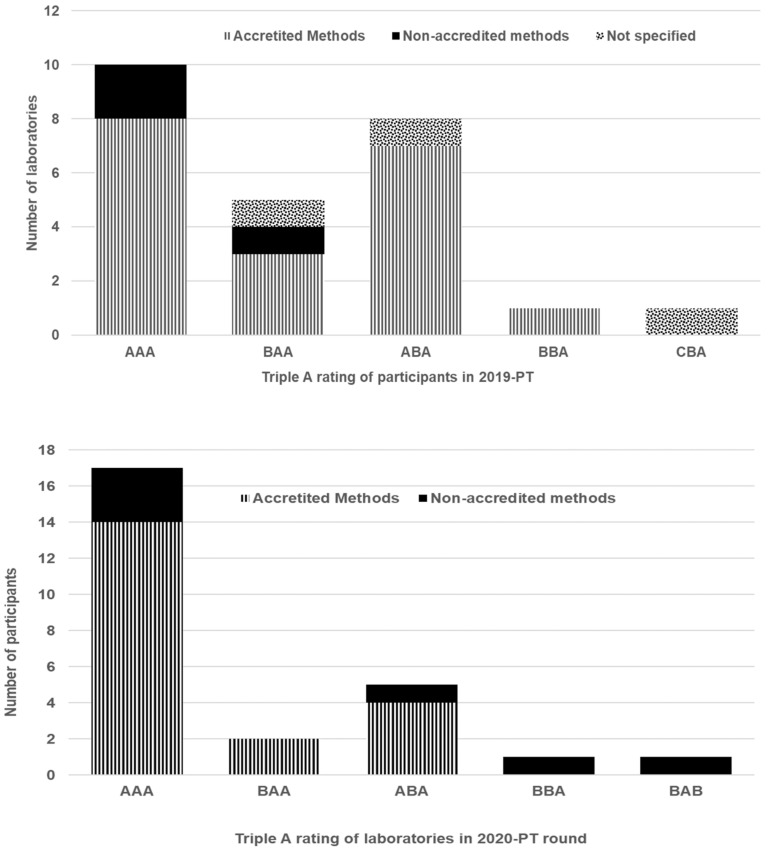
Laboratory performances according to the accreditation status of their analytical method.

Most of the participants used LC-MS/MS, but not all of the analytical methods were accredited. Based on the accreditation status of the analytical methods, the laboratory performances were evaluated for both of the PT rounds, as summarized in Figure 6.

Most analytical methods were accredited, being 76% (in 2019) and 77% (in 2020). The triple A rating showed that good and acceptable performances were achieved either with accredited or non-accredited analytical methods. It could be expected that laboratories using accredited methods would perform better, as their results are regularly subjected to third-party scrutiny [31]. It is worth mentioning that most of the non-accredited laboratories would be very much aware of the quality requirements in their own sector and may be working to standards that mimic accreditation. Non-accredited laboratories may well have accreditation as a goal for the near future. Moreover, they are trying to comply with the existing scheme’s performance criterion, as postulated by Thompson et al. [31].

### 2.6. Conclusion and Output

Efforts from some participants are needed in the estimation and reporting of the MU of their analytical method to allow the assignment of compliance towards the maximal accepted limits and the calculation of the ζ scores in inter-comparison laboratory trials.

For the participants, the provision of the PTs contributed to ascertaining and maintaining laboratory accreditation that is recognized all over the world.

The triple A approach seems to be adequate in evaluating the performance of laboratories participating in multi-mycotoxin PTs. Moreover, its combination with the sum of squared of z scores seems to be a sufficient evaluation tool for assessing performances.

Most of the analytical methods used in the PTs were accredited, but no clear differences were observed in the results obtained from the laboratories with or without accreditation.

Reference materials can be certified through this exercise and may increase the availability of such metrological tools, enabling the control of regulated mycotoxins throughout the world, pending additional long term stability monitoring.

## 3. Materials and Methods

### 3.1. Preparation of Candidate Reference Materials

In 2019, the test material was made of cornflakes, i.e., an unflavoured plain breakfast cereal product consisting of small toasted flakes of corn. In 2020, the candidate material was made of rusk, i.e., crispy and golden wheat bread. Both of these test materials were purchased in Belgian supermarkets.

The PT allowed the evaluation of the determination of the following toxins: AFB_1_, AFB_2_, AFG_1_, AFG_2_, OTA, FB_1_, FB_2_, DON, T2, HT2. In addition, three sums of mycotoxins were included: the sum of the fumosinins, the sum of the aflatoxins, and the sum of T2 and HT2 toxin.

The test materials were prepared in-house by mixing incurred batches with blank or contaminated filling flour. Mycotoxin contaminated materials were produced by inoculating and fermenting cereal grains with selected fungi [32]. The fermented materials were sterilized, dried, finely ground, and homogenized. Homogenization was carefully performed as described by Tangni et al. [23]. The bulk materials were divided by scale using a Retsch rotary sample divider PT100 to create individual subsamples (approximately 55.0 ± 0.5 g), which were dispensed into aluminum foil laminate sachets that were then vacuum sealed and numbered. Random sampling using computer-generated numbers was conducted to allocate the test materials for homogeneity testing, stability testing, and participant distribution. Surplus contingency samples were left for future use as a quality control reference material. All of the packed samples were stored at +4 °C prior to their distribution to the participant laboratories.

### 3.2. Homogeneity Testing

The homemade candidate materials were tested for homogeneity following the recommended procedures of the International Harmonized Protocol for the Proficiency Testing of Analytical Chemistry Laboratories [21]. The homogeneity data were statistically computed and assessed to ensure that any “questionable” and “unacceptable” laboratory performances could not be attributed to any significant sample variability of the PT materials.

Briefly, two test portions of twelve randomly selected samples were extracted and analyzed through liquid chromatography-tandem mass spectrometry (LC-MS/MS) under repeatability conditions (i.e., same laboratory, same analyst, same method, and same equipment) using the analytical ISO 17025-accredited method [23]. The data were tested for precision using analysis of variance to estimate the sampling and analytical variances. The test for “adequate” homogeneity was conducted as recommended by Fearn and Thompson [33] and the ISO 13528 [30].

### 3.3. Stability Testing

The short-term stability study of the targeted toxins in the materials was checked throughout the duration and ranged between sample distribution and the data submitting deadline (end of analyses, 10 weeks). A total of three test material sachets were randomly selected at the start of the dispatching, which were kept at −20 °C (used as reference temperature), +4 °C, and +24 °C (chosen as recommended and challenge temperatures, respectively). Mycotoxin analyses were performed in duplicate under repeatability conditions that were similar to those used for homogeneity testing [23]. The stability per analyte was evaluated by comparing the mean of the results at a given storage temperature and duration (Xi) with the mean of the results of the homogeneity testing or with the toxin loads determined at the start of stability experiment (X0), using the following criterion for σp [21].

(1) $$\left|\mathrm{Xi}-X0\right|\;\leq \;\mathrm{SQRT}\left[\left(0.3\times \sigma p\right)2\;+\;\left(0.5\times \sigma p\right)2\right]\;=\;0.6\times \sigma p$$

### 3.4. Proficiency Test Management

Based on ISO 17043, the technical inputs of each PT round were provided by an advisory board led by senior scientists from Sciensano. They were involved in the sample preparation, homogeneity and stability testing, administrative work (invitation, correspondences, shipping), statistical analyses, and reporting work according to the established time schedule. A draft report was submitted to the participants with the request to verify their reported data and to send their feedback comments within one month.

#### 3.4.1. Participants

Local governmental, private, academic, commercial food testing laboratories and industry quality control units around the world were invited to participate in the PT rounds. Invitations were sent by e-mail with the request to fill in the participation form mentioning the PT item, the analytes to be tested, the participation fee, and the timescale.

Each participant was given a laboratory number, which assigned according to the registration ranking. Laboratory codes were confidentially communicated to the corresponding participant and were used throughout the PT round to preserve the confidentiality of the provided results.

#### 3.4.2. Distribution of PT Items

Each participant received the receipt form to confirm the arrival, the test material samples, and an enclosed letter with instructions pertaining to sample handling. The reporting form (protected Excel^®^ file) was emailed after confirming the good status of the sample arrival. Each participant received one package (55.0 ± 0.5 g of sample), and reporting the results of the extraction and analysis of one sample was recommended. Results in µg/kg and the measurement uncertainty (MU_(k = 2)_) were reported. The participants were also asked to answer a few questions pertaining to the method(s) used.

#### 3.4.3. Applied Analytical Methods by Participants

Participant laboratories used their own routine analytical methods for the targeted mycotoxins. The test materials had to be re-homogenized before the routine analysis. The laboratories were given 6 weeks after the receipt of the PT items to finish the analyses with a warning of the deadline for results submission.

### 3.5. Statistical and Performance Evaluation

The assigned and uncertainties measurement values for mycotoxins in the PT materials were determined by the consensus of the participants’ results. The robust statistic approach is a convenient modern method of handling results when they are expected to follow a near-normal distribution. Medians of all results (MED_tot_) were calculated, and results ranging from 50% to 150% MED_tot_ were used as reference dataset. Otherwise, they were considered as outliers. The median of reference data set (MED_ref_) and median absolute difference (MAD) were used as robust estimators. Kernel density plots were built to check a convincing central tendency. A satisfactory HorRat value (<1.2) was used to assign the MED_ref_ as “consensus” or “reference” value for the targeted analyte. The modified Horwitz equation was used to establish the standard deviation for proficiency testing (σ_p_), calculated using the equation as described by Thompson [34].

#### 3.5.1. Single Analyte Performance Assessment

Individual laboratory performances were rated by a z score (Equation (2)) and ζ-score (Equation (3)) in accordance to the ISO procedure 13528 [30] and the International Harmonized Protocol described by Thompson et al. [21].

(2) $$z\;\mathrm{score}=\frac{\;X_{\mathrm{lab}}-V_{\mathrm{ass}}}{\sigma p}$$

(3) $$\zeta \;\mathrm{score}=(X_{\mathrm{lab}}-V_{\mathrm{ass}})/\sqrt{u_{ref}{}^{2}+u_{lab}{}^{2}}$$

where X_lab_ is the individual measurement result supplied by the participating laboratory, V_ass_ is the assigned value, σ_p_ is the standard deviation for proficiency assessment, *u_ref_* is the standard uncertainty for the assigned value, and *u_lab_* is the reported standard uncertainty on the reported value by the participating laboratory.

The z score compares the participant’s deviation from the reference value with regard to the standard deviation accepted for the proficiency test. The ζ score states if the laboratory result agrees with the assigned value within the uncertainty claimed by the laboratory. The z scores are interpreted as acceptable (│z│ ≤ 2), questionable (2 < │z│ < 3), and unacceptable (│z│ ≥ 3). Likewise, the ζ scores are also interpreted as acceptable when │ζ│ ≤ 2, questionable for 2 < │ζ│ < 3, and unacceptable when│ζ│ ≥ 3.

#### 3.5.2. Combined z Score Values Approach for Assessing Overall Laboratory Performance

Regarding multi-mycotoxin determination, several z score values are simultaneously generated and can be used to assess an overall performance via the sum of the squared z scores (SZ2, Equation (4)).

(4) $$\mathrm{SZ2}=\mathrm{SSZ}/n=\sum_{1}^{i}\frac{Zi^{2}}{n}$$

with SSZ = sum of squared z scores.

SZ2 has the advantage of maintaining the same classification thresholds of 2 and 3 as those used for single z score (e.g., (│z│ ≤ 2 = good performance; 2 < │z│ ≤ 3 = acceptable performance, and │z│ ≥ 3 = unacceptable performance). Therefore, SZ2 can be considered to facilitate a clearer and easier score differentiation obtained for the overall laboratory performance evaluation.

#### 3.5.3. Triple A Approach for Assessing Overall Laboratory Performance

The triple A approach is based on the ranking according to the number of analytes (*x*-axis), z score classification (*y*-axis), and the number of false positive results (*z*-axis) as follows [26]:

Number of analytes (e.g., A laboratory is classified as “A”, “B” or “C” when it reports the quantitative results “≥90%”, “<90% but ≥50%” or “<50%” of the analytes present in the test material, respectively): Laboratories classified as C in scope can only be classified as B or C in performance.

Ratio of acceptable z score (−2 ≤ z scores ≤ 2) (performance is defined by the ratio between the number of acceptable z scores obtained by the laboratory and the number of z score values assigned to the laboratory): The borderline criteria for establishing categories A, B and C are also the same as above. A laboratory is classified as “A”, “B” or “C” when it reports acceptable results “≥90%”, “<90% but ≥50%” or “<50%”, respectively, of the total z scores obtained by the laboratory.

False positive (using samples with a concentration < LOQ to check if the participant reported erroneous concentrations above the LOQs): Finally, laboratories are classified as “A”, “B”, or “C” when they report none, one, or more than one false positive, respectively [26].

## Figures and Tables

**Table 1:** Statistical evaluation for the homogeneity testing of cornflake flour (PT-2019).

	AFB_1_	AFG_1_	AFB_1_ + AFG_1_	DON	OTA	FB_1_	FB_2_	FB_3_	Sum FBs	T2	HT2	Sum (T2, HT2)
“Sufficient homogeneity”												
S_sam_	2.4	2.6	1.6	1.5	2.2	1.1	1.6	1.1	1.1	2.6	2.7	2.6
S_sam_/0.3σ_p_	0.4	0.34	0.2	0.3	0.3	0.2	0.2	0.2	0.2	0.4	0.4	0.4
S_sam_ < 0.3σ_p_	Pass	Pass	Pass	Pass	Pass	Pass	Pass	Pass	Pass	Pass	Pass	Pass
“Adequate homogeneity”												
σ^2^_all_ (µg/kg)	0.46	0.20	0.71	20.52	0.98	13.98	1.47	4.11	15.10	5.16	2.39	7.55
S_an_ (µg/kg)	0.70	0.30	0.70	17.96	1.34	9.06	1.22	2.45	10.05	7.55	2.96	10.14
c	0.81	0.15	1.34	1030.8	3.26	420.5	5.15	35.42	495.1	96.62	17.80	190.5
s_sam_/sqrt(c)	0.00	0.00	0.00	0.24	0.00	0.07	0.09	0.07	0.05	0.00	0.42	0.15
S^2^_sam_ < c	Pass	Pass	Pass	Pass	Pass	Pass	Pass	Pass	Pass	Pass	Pass	Pass

**Table 2:** Statistical evaluation for the homogeneity testing of rusk flour (PT-2020).

	AFB_1_	AFB_2_	AFG_1_	AFB_1_ + AFB_2_ + AFG_1_	DON	OTA	FB_1_	T2	HT2	Sum (T2 HT2)
“Sufficient homogeneity”										
S_sam_	1.9	2.7	1.8	1.8	4.0	2.4	1.9	1.1	2.0	1.2
S_sam_/0.3σ_p_	0.28	0.40	0.27	0.27	0.78	0.36	0.29	0.19	0.30	0.21
S_sam_ < 0.3σ_p_	Pass	Pass	Pass	Pass	Pass	Pass	Pass	Pass	Pass	Pass
“Adequate homogeneity”										
σ^2^_all_ (µg/kg)	0.83	0.04	0.43	1.26	34.15	0.48	1.89	18.11	4.55	21.40
S_an_ (µg/kg)	0.74	0.08	0.38	1.10	96.64	0.58	2.16	11.59	5.30	15.53
C										
s_sam_/sqrt(c)	0.30	0.00	0.21	0.26	0.00	0.26	0.00	0.13	0.00	0.13
S^2^_sam_ < c	Pass	Pass	Pass	Pass	Pass	Pass	Pass	Pass	Pass	Pass

c = critical value; S_an_^2^ = analytical variance, S^2^_sam_ = sampling variance, σ_p_ = fitness-for-purpose based standard deviation, σ^2^_all_ = allowed sampling variance. Homogeneity test: c = critical value = F1 × σ^2^_all_ + F2 × S^2^_an_ (if S^2^_sam_ < c), the test for homogeneity has been passed (F1 = 1.79 and F2 = 0.86).

**Table 3:** Results of stability testing in both test materials during the trial.

	Relative Changes * (Changes/0.6 × σ_p_)							
	Cornflakes				Rusk			
	Storage until End of the Dispatching Period at:		Storage until the End of the Result Delivery Period at:		Storage until End of the Dispatching Period at:		Storage until the End of the Result Delivery Period at:	
Mycotoxins	+4 °C	+24 °C	+4 °C	+24 °C	+4 °C	+24 °C	+4 °C	+24 °C
DON	−0.4	−0.3	0.1	−0.1	−0.57	−0.93	−0.71	−0.71
AFB_1_	−0.5	−0.6	−0.4	−0.5	−0.30	−0.52	−0.45	−0.85
AFB_2_	n.a.	n.a.	n.a.	n.a.	−0.11	−0.63	−0.53	−0.79
AFG_1_	−0.3	−0.5	−0.2	−0.5	n.a.	n.a.	n.a.	n.a.
T2	−0.4	−0.2	−0.3	−0.5	−0.26	−0.25	−0.22	−0.15
HT2	0.0	−1.3	0.9	0.5	−0.15	−0.32	−0.05	−0.22
FB_1_	−0.4	−0.2	−0.3	−0.5	−0.35	−0.33	−0.21	−0.22
FB_2_	−0.2	0.1	−0.2	−0.6	n.a.	n.a.	n.a.	n.a.
FB_3_	−0.5	−0.2	−0.4	−0.8	n.a.	n.a.	n.a.	n.a.
OTA	−0.3	0.3	−0.6	−0.5	−0.21	−0.31	−0.75	−0.55

*: Significant mycotoxin decrease occurred when negative relative changes were above 1.0; (SQRT [(0.3 × σ_p_)^2^ + (0.5 × σ_p_)^2^] = 0.6σ_p_ [21]; n.a: not applicable.

**Table 4:** Summary of statistical evaluations for the test materials and for laboratory performances (2019).

	AFB_1_	AFB_2_	AFG_1_	AFG_2_	Sum AFs	DON	FB_1_	FB_2_	FB_1_ + FB_2_	FB_3_	OTA	T2	HT2	T2 + HT2
Calculations—Uncensored dataset														
n_tot_	24	19	23	13	21	25	23	22	21	10	24	24	23	22
MED_tot_ (µg/kg)	6.2	0.5	2.7	0.2	9.2	380.0	223.3	26.1	245.4	54.7	13.5	73.8	35.5	107.0
MAD_tot_ (µg/kg)	0.2	0.1	0.4	0.1	1.0	32.7	45.8	4.4	47.6	6.8	1.6	9.5	4.5	19.5
Calculations—Reference dataset														
n_ref_	24	15	23	10	21	22	23	17	21	10	23	22	21	19
MED_ref_ (µg/kg)	6.2	0.5	2.7	0.3	9.2	376.6	223.3	25.7	245.4	54.7	13.5	73.8	35.5	105.9
MAD_ref_ (µg/kg)	0.2	0.1	0.4	0.1	1.0	29.0	45.8	3.3	47.6	6.8	1.5	8.6	4.5	17.8
σ_rob_ (µg/kg)	0.4	0.1	0.6	0.1	1.5	43.0	67.9	4.9	70.6	10.0	2.1	12.7	6.7	26.4
σ_p_ (µg/kg)	1.4	0.1	0.6	0.1	2.0	69.8	44.8	5.7	48.5	12.0	3.0	16.2	7.8	23.3
HorRat	0.3	1.3	1.0	1.3	0.7	0.6	1.5	0.9	1.5	0.8	0.7	0.8	0.9	1.1
V_ass_ (µg/kg)	6.2	-	2.7	-	9.2	376.6	-	25.7	-	54.7	13.5	73.8	35.5	105.9
u (µg/kg)	0.1	-	0.1	-	0.3	9.2	-	1.2	-	3.2	0.4	2.7	1.5	6.1
Z score results														
n_tot_	24		23		21	25		22		10	24	24	23	22
Acceptable	100.0%		100.0%		100.0%	88.0%		77.3%		100%	95.8%	87.5%	91.3%	86.4%
Questionable	0.0%		0.0%		0.0%	0.0%		9.1%		0%	0.0%	4.2%	0.0%	9.1%
Unacceptable	0.0%		0.0%		0.0%	12.0%		13.6%		0%	4.2%	8.3%	8.7%	4.5%
Zeta score results														
n_tot_	21		20		15	21		18		8	18	21	20	13
Acceptable	90.5%		90.0%		86.7%	81.0%		88.9%		88%	94.4%	85.7%	75.0%	100.0%
Questionable	0.0%		0.0%		0.0%	0.0%		0.0%		13%	0.0%	4.8%	0.0%	0.0%
Unacceptable	9.5%		10.0%		13.3%	19.0%		11.1%		0%	5.6%	9.5%	25.0%	0.0%

Assigned values V_ass_ are the medians of the reference results; σ_p_: standard deviation for proficiency assessment. The uncertainties of the consensus values did not exceed 0.7 × σ_p_, so statistical evaluation is appropriate. ND: non detected, level below limit of detection; *: no statistical assessment available.

**Table 5:** Summary of statistical evaluations for the test materials and for laboratory performances (2020).

	AFB_1_	AFB_2_	AFG_1_	Sum AFs	DON	FB_1_	OTA	T2	HT2	T2 + HT2
Calculations—Uncensored dataset										
n_tot_	26	21	22	22	26	20	25	26	24	23
MED_tot_ (µg/kg)	12.8	0.6	6.7	20.4	574.0	34.7	8.4	364.9	77.8	439.7
MAD_tot_ (µg/kg)	0.97	0.08	1.1	2.1	65.0	4.85	1.9	47.9	8.2	49.3
Calculations—Reference dataset										
n_ref_	26	20	21	22	26	19	23	25	20	22
MED_ref_ (µg/kg)	12.8	0.6	6.8	20.4	574.0	33.4	8.4	360.0	76.5	437.4
MAD_ref_ (µg/kg)	1.0	0.1	1.0	2.1	65.0	4.8	1.8	41.0	7.6	41.8
σ_rob_ (µg/kg)	1.4	0.1	1.5	3.1	96.4	7.1	2.7	60.8	11.2	61.9
σ_p_ (µg/kg)	2.8	0.1	1.5	4.5	99.8	7.3	1.8	67.2	16.8	79.2
HorRat	0.5	0.7	1.0	0.7	1.0	1.0	1.0	0.9	0.7	0.8
V_ass_ (µg/kg)	12.8	0.6	6.8	20.4	574.0	33.4	8.4	360.0	76.5	437.4
u (µg/kg)	0.28	0.02	0.32	0.66	18.9	1.6	0.6	12.2	2.5	13.2
Z score results										
n_tot_	26	21	22	22	26	20	25	26	24	23
Acceptable	100.0%	90.5%	90.9%	100.0%	92.3%	90.0%	84.0%	96.2%	79.2%	91.3%
Questionable	0.0%	4.8%	9.1%	0.0%	7.7%	10.0%	8.0%	0.0%	0.0%	4.3%
Unacceptable	0.0%	4.8%	0.0%	0.0%	0.0%	0.0%	8.0%	3.8%	12.5%	4.3%
Zeta score results										
n_tot_	25	20	21	17	25	19	24	25	23	16
Acceptable	88.0%	70.0%	71.4%	88.2%	84.0%	94.7%	83.3%	84.0%	82.6%	75.0%
Questionable	12.0%	20.0%	9.5%	11.8%	12.0%	0.0%	4.2%	8.0%	4.3%	6.3%
Unacceptable	0.0%	10.0%	19.0%	0.0%	4.0%	5.3%	12.5%	8.0%	13.0%	18.8%

Assigned values Vass are the medians of the reference results; σ_p_: standard deviation for proficiency assessment. The uncertainties of the consensus values did not exceed 0.7 × σ_p_, so statistical evaluation is appropriate. ND: non detected, level below limit of detection; *: no statistical assessment available.

**Table 6:** Results of triple A rating according to SZ2 classification over two PTs.

	SZ2 Evaluation	Triple A Rating						Overall
		AAA	BAA	ABA	BBA	CBA	BAB	
2019-PT	Good	11 (44%)	5 (20%)	0	0	0	0	16 (64%)
	Acceptable	0	0	4 (16%)	0	0	0	4 (16%)
	Unacceptable	0	0	3 (12%)	1 (4%)	1 (4%)	0	5 (20%)
2020-PT	Good	18 (69%)	2 (8%)	1 (4%)	0	0	1 (4%)	22 (85%)
	Acceptable	0	0	1 (4%)	0	0	0	1 (4%)
	Unacceptable	0	0	2 (7%)	1 (4%)	0	0	3 (11%)
Combined Rounds	Good	29 (57%)	7 (13%)	1 (2%)	0	0	1 (2%)	38 (74%)
	Acceptable	0	0	5 (10%)	0	0	0	5 (10%)
	Unacceptable	0	0	5 (10%)	2 (4%)	1 (2%)	0	8 (16%)

Good: Good performance; Acceptable: acceptable performance; Unacceptable: unacceptable performance.
